# Supplementary material for: An Instrumented Golden Eagle’s (Aquila chrysaetos) Long-Distance Flight Behavior
Source: Animals (Basel). 2022 Jun 6;12(11):1470. doi: 10.3390/ani12111470 (PMC9179650; doi:10.3390/ani12111470)
Supplement: Supplementary file 1 [file animals-12-01470-s001.zip › animals-1707319-supplementary.pdf]

Supplementary Table S1 Surface weather reports from NWS stations at a) Grant county Airport, WV and b) Cumberland, MD on 24 March 2016.

a)

Date/Time	Temp (^oF)	RH (%)	Sky	Vis (miles)	WD (deg)	WS (m/s)
3-24-2016 0:55:00	40	81	CLR:00	10.00	250	3
3-24-2016 1:55:00	37	86	CLR:00	10.00	000	0
3-24-2016 2:55:00	38	87	CLR:00	10.00	000	0
3-24-2016 3:55:00	37	87	CLR:00	10.00	000	0
3-24-2016 4:55:00	36	88	CLR:00	10.00	000	0
3-24-2016 5:55:00	35	92	CLR:00	10.00	000	0
3-24-2016 6:55:00	35	91	CLR:00	10.00	000	0
3-24-2016 7:55:00	42	80	CLR:00	10.00	000	0
3-24-2016 8:55:00	50	64	CLR:00	10.00	000	0
3-24-2016 9:55:00	57	47	CLR:00	10.00	000	0
3-24-2016 11:15:00	72	40	CLR:00	10.00	130	10
**3-24-2016 11:55:00****	**74**	**35**	**CLR:00**	**10.00**	**220**	**8**
**3-24-2016 12:55:00****	**76**	**33**	**CLR:00**	**10.00**	**170**	**9**
**3-24-2016 13:55:00****	**78**	**31**	**CLR:00**	**10.00**	**190**	**10**
3-24-2016 14:55:00	79	29	CLR:00	10.00	210	14
3-24-2016 15:55:00	78	30	CLR:00	10.00	210	14
3-24-2016 16:55:00	78	31	SCT:04 90	10.00	190	16
3-24-2016 17:55:00	75	35	CLR:00	10.00	160	13

** - The time of the Eagle flight. 16UTC is 12:00 LST

b)

Date/Time	Temp (^oF)	RH (%)	Sky	Vis (miles)	WD (deg)	WS (m/s)
3-24-2016 0:55:00	50	54	CLR:00	10.00	360	5
3-24-2016 1:55:00	46	66	CLR:00	10.00	000	0
3-24-2016 2:55:00	45	71	CLR:00	10.00	000	0
3-24-2016 3:55:00	43	81	CLR:00	10.00	000	0
3-24-2016 4:55:00	43	81	CLR:00	10.00	000	0
3-24-2016 5:55:00	41	81	CLR:00	10.00	000	0
3-24-2016 6:55:00	41	87	CLR:00	10.00	000	0
3-24-2016 7:55:00	45	76	CLR:00	10.00	000	0
3-24-2016 8:55:00	54	58	CLR:00	10.00	000	0
3-24-2016 9:56:00	59	45	CLR:00	10.00	170	3
3-24-2016 10:56:00	66	32	CLR:00	10.00	210	5
**3-24-2016 11:56:00****	**72**	**29**	**CLR:00**	**10.00**	**130**	**8**
**3-24-2016 12:56:00****	**77**	**28**	**CLR:00**	**10.00**	**VRB**	**11**
**3-24-2016 13:56:00****	**79**	**24**	**CLR:00**	**10.00**	**160**	**9**
3-24-2016 14:56:00	79	24	CLR:00	10.00	150	14
3-24-2016 15:56:00	77	28	CLR:00	10.00	150	20
3-24-2016 16:56:00	77	28	CLR:00	10.00	170	17
3-24-2016 17:57:00	75	29	CLR:00	10.00	160	16

** - The time of the Eagle flight. 16UTC is 12:00 LST

Supplementary Table S2. Clockwise circling closed circles numbered (ID) in sequence at a specific time (UTC: hr, min, sec). Dwell is time(s), Height is starting altitude (m) and Z is height gain (m) in the circle.

Clockwise Circling						
**ID**	**Hour**	**Min**	**Sec**	**Dwell(s)**	**Height(m)**	**Z gain (m)**
1	15	59	39	14	692.2	11
2	15	59	53	18	699.7	3
4	16	02	24	9	798.4	14
5	16	02	34	17	816.6	30
6	16	02	52	18	851	30
7	16	03	09	12	883.9	27
8	16	03	21	16	915.4	37
9	16	03	39	17	959.6	30
10	16	03	55	17	991.2	32
11	16	04	11	21	1025.2	30
12	16	04	32	18	1057	34
13	16	04	51	20	1093	35
14	16	05	11	16	1128.6	29
15	16	5	27	15	1158	35
17	16	06	56	36	1358.9	0
21	16	10	32	32	1478.5	46
22	16	11	08	22	1528.9	18
24	16	12	10	17	1626	26
29	16	14	19	16	1932.3	24
36	16	29	49	17	1823.9	52
41	16	42	43	23	1549.1	58
42	16	43	07	18	1622.8	73
44	16	51	05	28	1530.6	49
45	16	51	35	22	1587.7	29
46	16	51	58	25	1620.5	56
47	16	52	25	25	1681.4	40
50	16	58	13	28	1727.9	46
51	17	01	53	24	1639.6	61
56	17	03	42	16	1877.4	43
58	17	08	26	25	2014.2	52
59	17	08	56	21	2083.7	34
60	17	09	23	21	2129.3	30
67	17	28	25	24	1556.7	46
68	17	28	49	17	1604.1	34
71	17	36	38	23	1453.6	30
73	17	37	44	22	1578.7	40
74	17	39	24	20	1745.7	43
77	17	42	57	15	1833.2	30
78	17	43	13	26	1868.7	30
79	17	43	39	20	1903.4	30
81	17	49	26	27	1887.2	58
82	17	51	48	19	2214.5	53
84	17	57	35	23	2146.6	49

Supplementary Table S3. As for Table S2 but for counterclockwise circling.

Counterclockwise Circling						
**ID**	**Hour**	**Min**	**Sec**	**Dwell(s)**	**Height(m)**	**Z gain (m)**
3	16	01	57	13	767	20
4	16	2	10	14	782.2	48
15	16	5	42	13	1196	33
16	16	05	58	24	1231	61
18	16	09	04	32	1316.3	46
19	16	09	36	25	1362	46
20	16	10	01	26	1413.7	55
23	16	11	41	26	1581.7	26
25	16	12	38	23	1668.1	30
26	16	13	11	16	1715.7	46
27	16	13	31	17	1782.1	43
28	16	13	57	11	1849.2	37
30	16	18	16	26	1629.4	12
31	16	19	41	30	1697.9	34
32	16	23	28	28	1723	53
33	16	23	57	30	1779.7	64
34	16	24	28	23	1846.9	11
35	16	25	19	28	1905.2	15
37	16	30	06	18	1881.5	46
38	16	30	25	22	1932.2	30
39	16	30	56	23	1980.2	43
40	16	31	21	20	2034.5	40
43	16	44	35	19	1883.2	37
48	16	54	56	26	1712.2	61
49	16	55	23	29	1777.2	79
52	17	02	15	15	1697.4	27
53	17	02	33	19	1733.4	49
54	17	02	51	28	1783.2	61
55	17	03	19	23	1848.1	24
57	17	07	38	22	1897.8	46
61	17	21	48	18	1450.1	15
62	17	25	34	23	1309.3	61
63	17	26	00	29	1382.2	61
64	17	26	29	28	1445.3	38
65	17	26	58	25	1490.3	24
66	17	27	26	25	1514.8	18
69	17	35	36	33	1377.2	46
70	17	36	09	28	1427.5	29
72	17	37	18	23	1516.9	53
75	17	40	10	20	1842.4	40
76	17	42	41	16	1838.9	-6
80	17	48	43	31	1780.5	69
83	17	56	51	27	2073.9	24
85	17	58	05	27	2223.5	15

Supplementary Table S4. Open circles identified as "meandering" with remainder as for Table S2.

**Meandering**						
**ID**	**Hour**	**Min**	**Sec**	**Dwell(s)**	**Height (m)**	**Z gain (m)**
1	15	59	01	38	679.3	15
2	16	00	11	30	702.8	15
3	16	00	41	76	716.9	46
4	16	06	21	35	1292	61
5	16	20	11	83	1732	13
6	16	28	58	25	1642.1	91
7	16	29	23	26	1735.3	84
8	17	39	00	24	1685.5	76

Supplementary Table S5. Linear gliding flight with average ground speed and direction with remainder as for Table S2.

**Gliding: Ascending and Descending**								
**ID**	**Hour**	**Min**	**Sec**	**Dwell (s)**	**Height (m)**	**Z gain (m)**	**Ground Speed (m/s)**	**Direction**
1	15	57	46	65	737	-57	10.5	264
2	16	07	33	82	1357.8	-38	16	31
3	16	11	29	11	1551.2	28	15.1	44
4	16	14	35	139	1956.7	-331	13.7	Var
5	16	18	41	51	1644.5	55	11.2	Var
6	16	20	11	169	1732.3	-54	18.9	75
7	16	24	50	31	1859	44	15.4	36
8	16	25	48	169	1920.9	-280	19.7	51
9	16	31	41	573	2072.2	-689	18.7	53
10	16	46	05	251	2011.8	-518	19.7	42
11	16	53	54	54	1705.4	6	18.7	22
12	16	55	51	109	1857.5	-147	20.2	28
13	16	58	40	145	1771.9	-137	19.8	37
14	17	03	59	183	1928.1	-50	17.8	24
15	17	07	59	23	1947.9	64	15.4	358
16	17	09	44	622	2158.9	-712	20.1	23
17	17	22	05	183	1465.5	-158	19.9	12
18	17	27	51	31	1541	13	16.7	56
19	17	29	06	217	1637.7	-392	22.4	22
20	17	37	00	15	1486.9	28	16.1	19
21	17	38	05	45	1618.3	53	17.5	23
22	17	39	44	20	1785.7	53	15.6	41
23	17	40	31	116	1884.9	-49	21.2	22
24	17	43	59	244	1940.8	-164	19.9	27
25	17	51	15	28	2153.3	57	15.4	45
26	17	52	06	244	2270.1	-196	21	39
27	17	58	33	301	2239.7	-826	25	20

Supplementary Table S6. Detailed summary of small-scale flight maneuvers used during flight (CC = counterclockwise and C = clockwise).

	CC Circle	C Circle	Meander	Glide Up	Glide Down
Number of classified segments	44	42	8	10	17
Average segment time (s)	23.2	20.5	42.1	35	267.7
Standard deviation (s)	5.6	5.3	23.6	17	183.6
Minimum time (s)	11	9	24	12	74
Maximum time (s)	33	36	83	64	723
Average height change (m)	38.7	36.3	50.1	40	-282
Standard deviation (m)	18.4	15.2	32.8	20	258
Minimum height change (m)	-6.1	0	12.5	6	-38
Maximum height change (m)	79.3	73.2	91.5	64	-826
Average climb/descent rate (m/s)	1.7	1.8	1.7	1.4	-1
Standard deviation (m/s)	0.8	0.7	1.5	0.8	0.54
Maximum descent rate (m/s)	-0.4	0	0.1	N/A	-2.3
Maximum climb rate (m/s)	3.43	4.1	3.7	2.4	N/A
Average ground speed (m/s)	n/a	n/a	n/a	15.7	19.1
Standard deviation (m/s)	n/a	n/a	n/a	1.95	3.3
Minimum average ground speed (m/s)	n/a	n/a	n/a	11.2	10.5
Maximum average ground speed (m/s)	n/a	n/a	n/a	18.7	25
Total time in maneuver (min)	17	14.4	5.6	5.8	75.8
Percent total time*	14.0	11.9	4.6	4.8	62.6
Net height change for maneuver (m)	1702.8	1524.6	400.8	400.0	-4794.0

*Not including missing or unclassified segments totaling 7.7 minutes.

a)

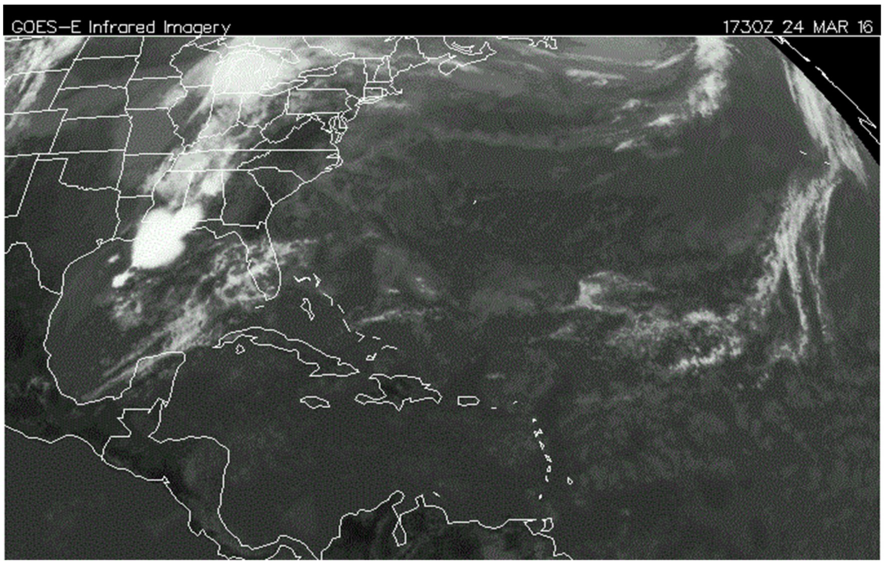

b)

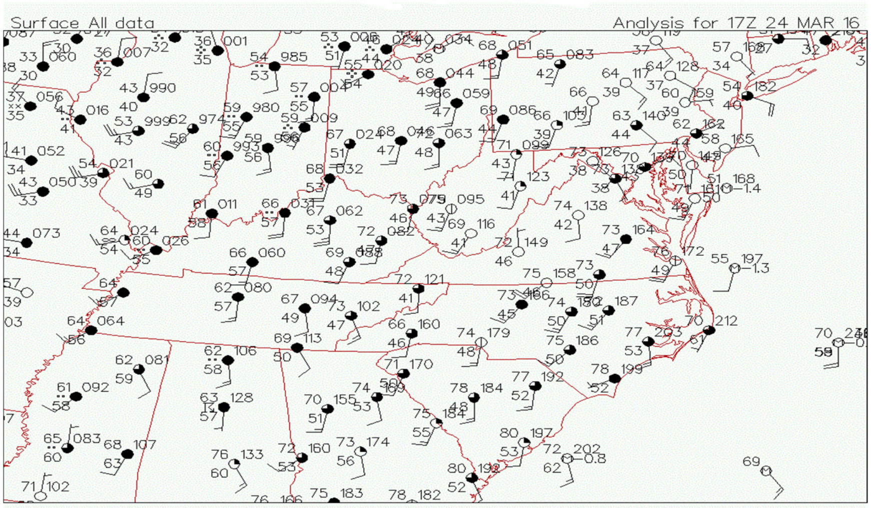

Supplementary Figure S1: GOES IR satellite imagery for 1730 UTC, 24 March 2016 (image courtesy of Plymouth State University Weather Center) (Figure S1a) and surface weather plot for 1730 UTC, 24 March 2016 (Figure S1b). Open circle means clear conditions. Weather station plots are standard synoptic symbols: top left – temperature (°F), top right – pressure in millibars; bottom right – dewpoint (°F); barb – wind direction feathers full 10 m/s; half – 5 m/s, shading (circle) degrees of sky cloud cover (image courtesy of Plymouth State University Weather Center).

a)

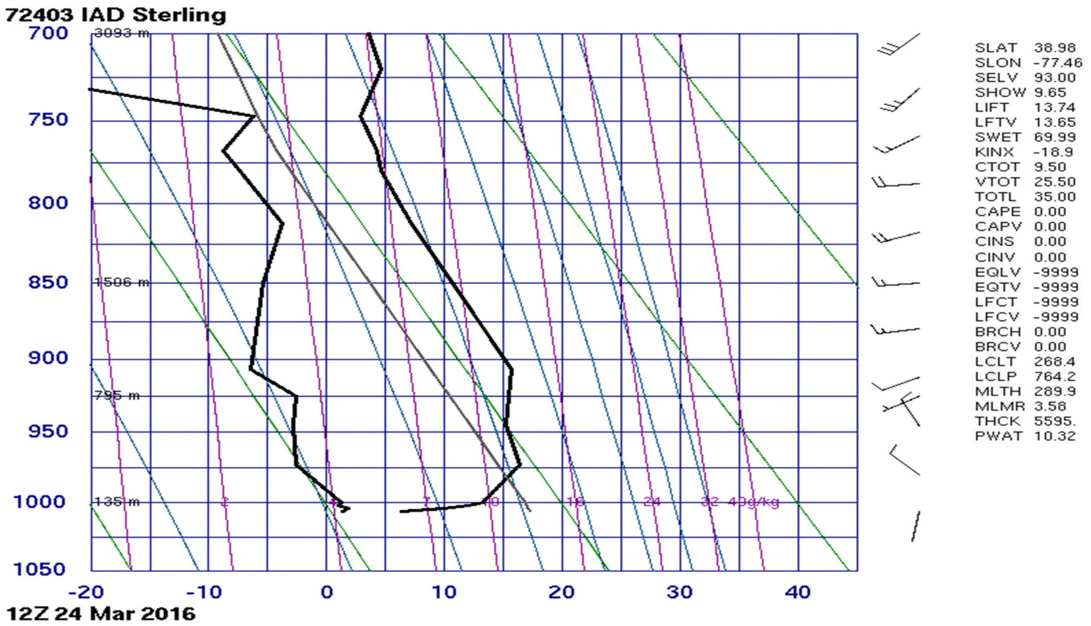

b)

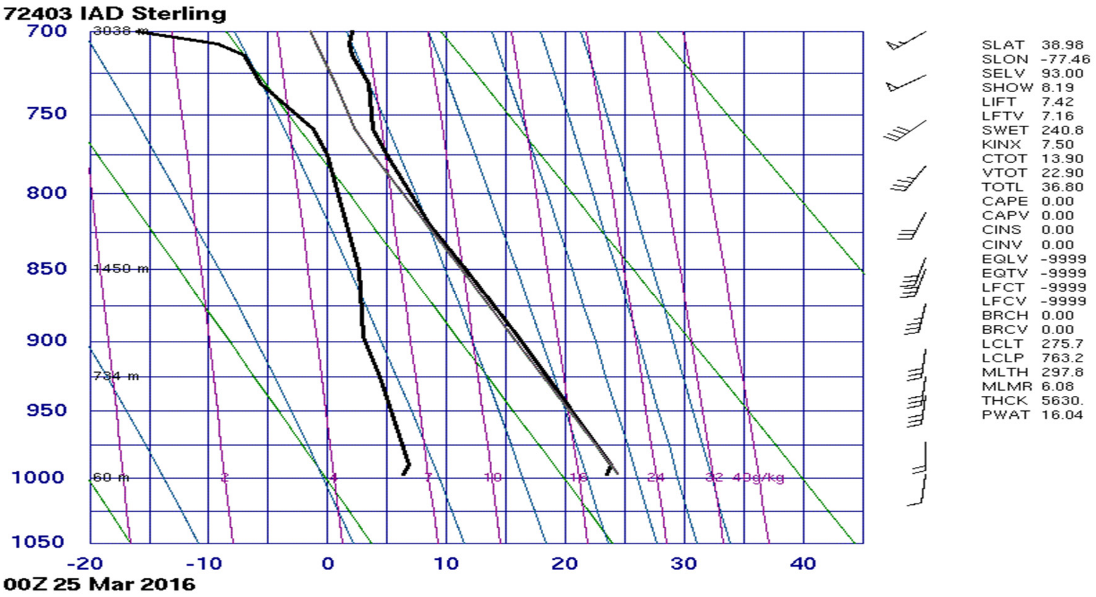

Supplementary Figure S2: Vertical sounding of temperature specific humidity and wind (right Hand side) for Dulles (IAD) at 1200 UTC on 24 March 2016 (a) and 0000 UTC on 25 March 2016 (b).

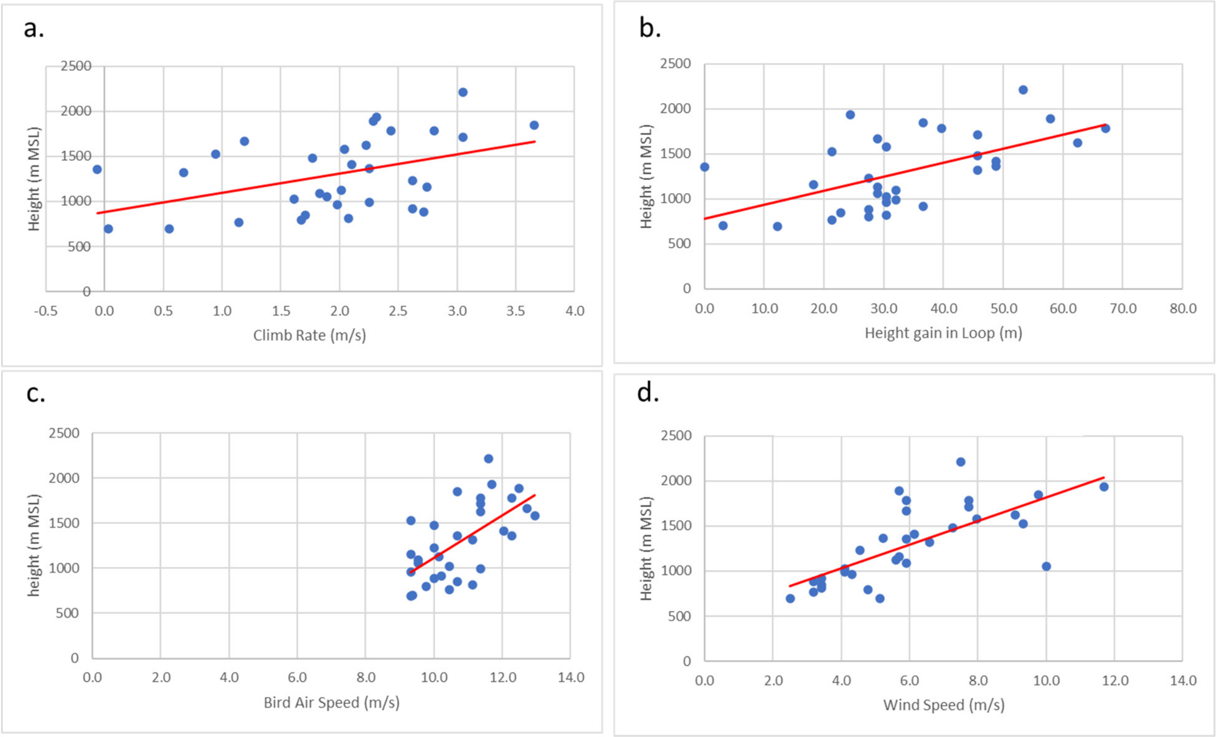

Supplementary Figure S3. Quantities derived from GPS data during the initial ascent from surface to 1900 m (Fig. 1). a) Eagle climb rate (m s^{-1}) vs height (m ASL). Average climb rate is 1.9 m s^{-1}. b) Height gain (m) for individual circles. Average height gain is 30.6 m. c) True Air Speed (TAS) (m s^{-1}) of the eagle within each circle as a function of height (m ASL).
d) Horizontal wind speed derived from circles vs height (m ASL). Average shear = $9.1*10^{-3}$ s^{-1}.

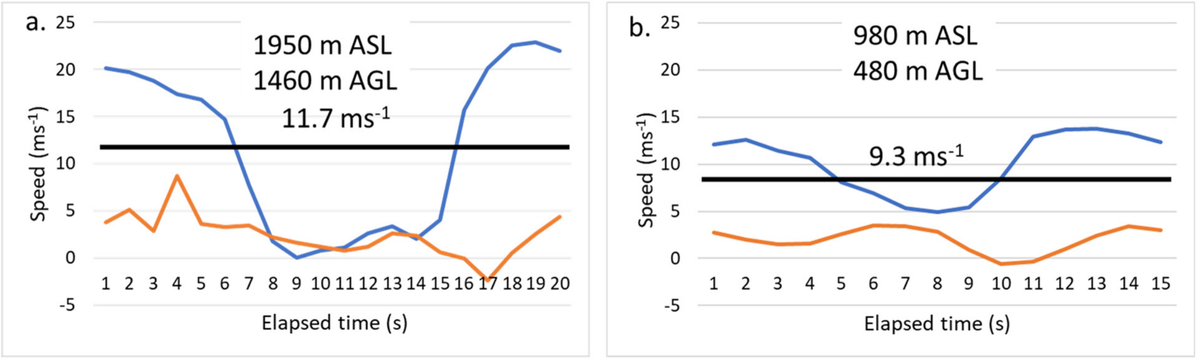

Supplementary Figure S4. Examples of the eagle's ground (blue line) and vertical speed (orange line) during circling: (a) circle near 1950 m ASL (1460 m AGL) with a mean ground speed (black line) of 11.7 m s^{-1} and (b) circle near 980 m ASL (480 m AGL) with a mean groundspeed of 9.3 m s^{-1}.

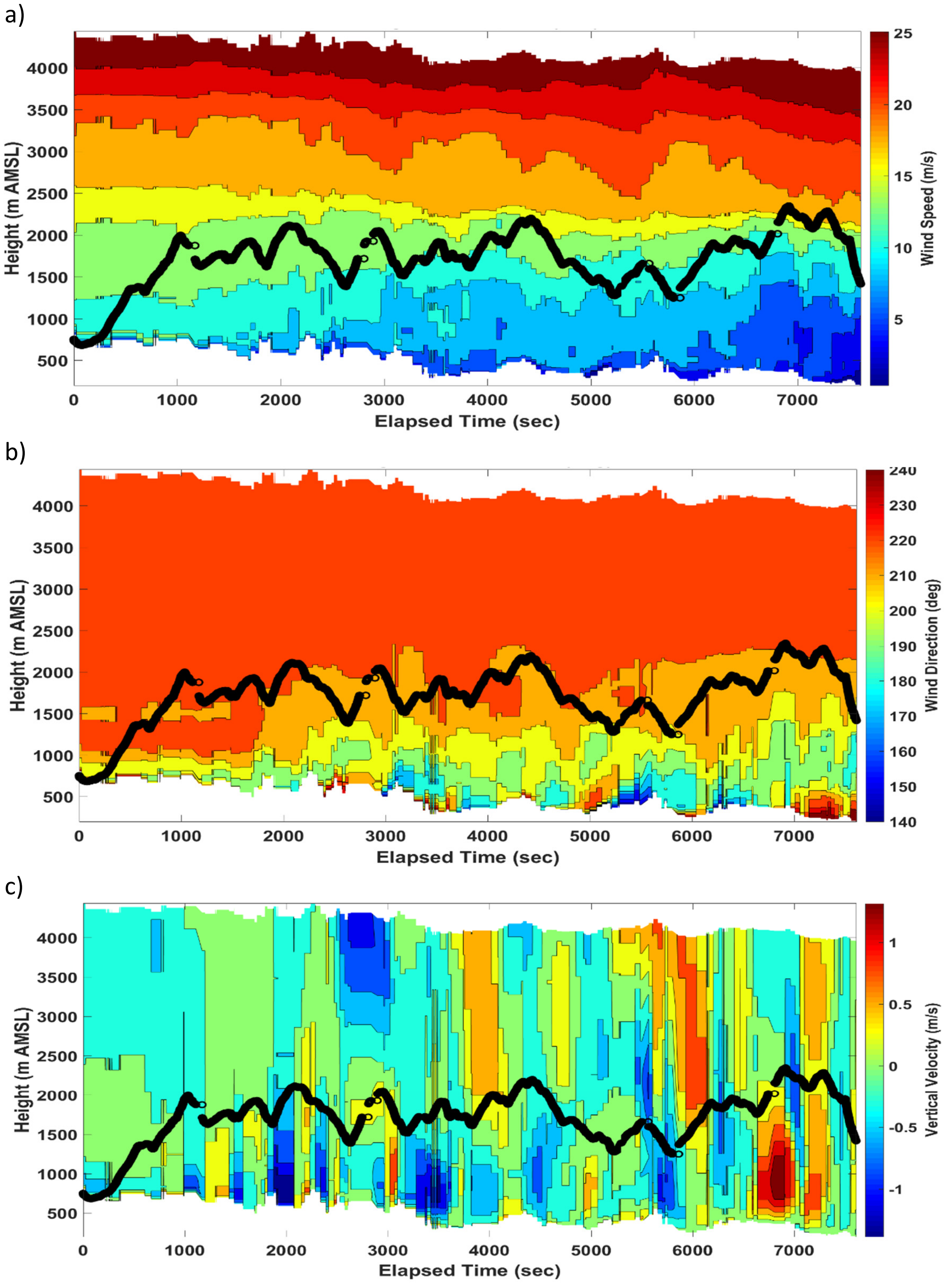

Supplementary Figure S5. WRF model Domain 3 (1 km) cross-sections of (a) wind speed, (b) wind direction and (c) vertical velocity profiles at 17 UTC along the flight path of the Golden Eagle . For each eagle waypoint, the nearest WRF model grid point profiles at 17 UTC were used.

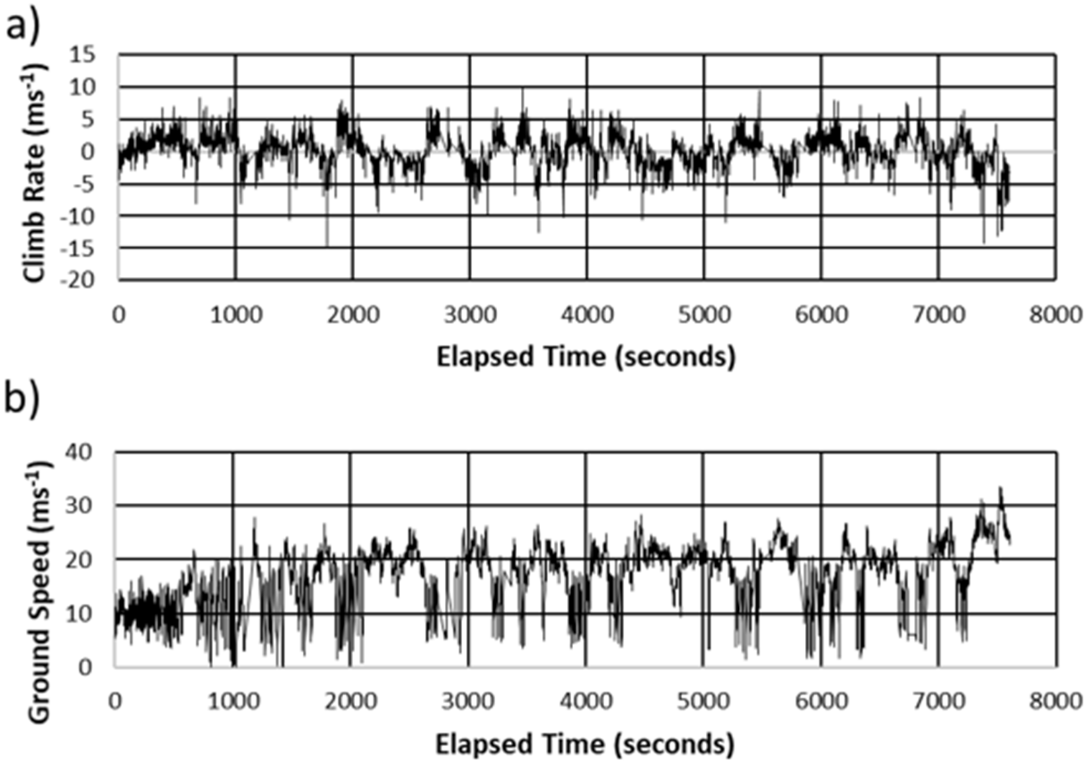

Supplementary Figure S6. (a) Eagle's climb rate in m s^{-1}; and (b) ground speed in m s^{-1} over the entire 103.5 km eagle flight. Note: These rates assume the eagle is always flying at the optimal glide speed with a sink rate of -.75 m s^{-1} and the sink rate has been accounted for in figure 6a and the values plotted include the required upward motions of the atmosphere.
